# Comparative Study of Cutaneous Squamous Cell Carcinogenesis in Different Hairless Murine Models

**DOI:** 10.3390/cancers16203546

**Published:** 2024-10-21

**Authors:** Georgios Gkikas, Dimitrios Katsiris, Andreas Vitsos, Anna Gioran, Dimitra Ieronymaki, Maria Kostaki, Georgios Ladopoulos, Vaya Ioannidou, Elisavet Theodoraki, Niki Chondrogianni, Ioannis Sfiniadakis, Georgios T. Papaioannou, Michail Christou Rallis

**Affiliations:** 1 Section of Pharmaceutical Technology, Department of Pharmacy, School of Health Sciences, National and Kapodistrian University of Athens, Panepistimiopolis Zografou, 15784 Athens, Greece; georgegikas1997@gmail.com (G.G.); djkatsiris@gmail.com (D.K.); avitsos@pharm.uoa.gr (A.V.); dimitraier93@gmail.com (D.I.); marie.kstk@gmail.com (M.K.); lgeorge4@hotmail.com (G.L.); ioannidou.vaya@gmail.com (V.I.); elisavet.theodor@gmail.com (E.T.);; 2 National Hellenic Research Foundation, Institute of Chemical Biology, 48 Vassileos Constantinou Ave., 11635 Athens, Greece; agioran@eie.gr (A.G.); nikichon@eie.gr (N.C.); 3 Pathologoanatomic Laboratory, Naval Hospital of Athens, 11521 Athens, Greece; jsfiniadakis@yahoo.gr

**Keywords:** cutaneous squamous cell carcinoma, UV radiation, photo-carcinogenesis, hairless mouse models, skin tumors

## Abstract

**Background**: In recent decades, a significant global increase in the incidence of non-melanoma skin cancer has been observed. To explore the pathogenesis of and potential therapeutic approaches for squamous cell carcinoma, various in vivo studies using mouse models have been conducted. However, investigations comparing different hairless mouse models, with or without melanin, as well as models with hypercholesterolemia and immunosuppression, in terms of their ability to induce squamous cell carcinoma have yet to be undertaken. **Methods**: Four mouse strains, namely SKH-hr1, SKH-hr2, SKH-hr2+ApoE, and immunodeficient Nude (Foxn1 knockout), were exposed to UVA and UVB radiation three times per week, initially to 1 Minimal Erythemal Dose (MED), incrementally increased weekly to a maximum dose of 3 MED. Clinical evaluation, photodocumentation, and biophysical parameters were monitored, along with proteasome protein activity and histopathological assessments. **Results**: The SKH-hr1 model primarily developed actinic keratosis without significant progression to invasive squamous cell carcinoma (SCC), while the SKH-hr2 and SKH-hr2+ApoE models exhibited a higher likelihood and intensity of papilloma and aggressive SCC formation, with the latter showing upregulated proteasome activity. Histopathological analysis confirmed the presence of poorly differentiated, invasive SCCs in the SKH-hr2 and SKH-hr2+ApoE models, contrasting with the less aggressive SCCs in the Nude mice and the mixed lesions observed in the SKH-hr1 mice. **Conclusions**: The SKH-hr2+ApoE and SKH-hr2 mice were identified as the most suitable for further exploration of squamous cell carcinogenesis. In contrast, the SKH-hr1 mice were found to be the least suitable, even though they are albino. Notably, proteasome analysis revealed a potential role of proteasome activity in squamous cell carcinogenesis.

## Simple Summary

Animal models are crucial for exploration of the causes of and potential treatments for squamous cell carcinoma, a common type of skin cancer. This study compared four different hairless mouse models to determine which is most suitable for studying skin cancer development when exposed to ultraviolet (UV) light. The models varied in their skin characteristics, such as the presence of melanin, cholesterol level, and immune system function. By monitoring various skin health parameters and analyzing proteasome activity, this study found that the SKH-hr2+ApoE and SKH-hr2 models were the most effective for further research into squamous cell carcinoma. In contrast, the SKH-hr1 model, despite its common use, was less suitable. These findings will help guide future research efforts in understanding and developing treatments for skin cancer.

## 1. Introduction

Skin cancer is the most common type of cancer, especially in populations with light-colored skin [1]. Skin cancers are divided into melanoma and non-melanoma skin cancers (NMSCs), in which basal and squamous cell carcinoma are included [2]. Cutaneous squamous cell carcinoma (cSCC) ranks as the second most prevalent skin cancer in the United States, following basal cell carcinoma [3]. It originates from precursor lesions known as actinic keratosis and can progress to metastasis. The main risk factor for developing cSCC is ultraviolet (UV) solar radiation, with lifetime cumulative exposure being a critical determinant [4]. Treatment primarily involves surgical excision, while radiation therapy is an option for some patient groups [5]. Immunosuppression elevates the risk of cSCC, and while metastasis in sun-exposed areas is rare, it poses a greater threat to immunocompromised individuals [6].

Ultraviolet radiation has been established as the primary environmental risk factor for cutaneous squamous cell carcinoma (cSCC), primarily through its ability to induce direct DNA damage. UVB radiation, in particular, is responsible for the formation of cyclobutane pyrimidine dimers (CPDs) in the DNA of keratinocytes, which are the most common photoproducts caused by UV exposure. These CPDs form when UVB photons induce covalent bonds between adjacent pyrimidine bases, leading to the creation of bulky lesions in the DNA strand [7]. When left unrepaired by the nucleotide excision repair system, these lesions can result in mutations, which are characteristic “UV signature” mutations. These mutations frequently occur in tumor suppressor genes like TP53, which is found to be mutated in up to 95% of human metastatic cSCC cases [8].

On the other hand, UVA radiation, although less efficient at causing direct DNA mutations, penetrates deeper into the skin and contributes to the cumulative damage that can drive carcinogenesis by causing oxidative stress and promoting mutations over time. It is noteworthy that indirect DNA damage plays a significant role in UV-mediated cSCC development, especially through the actions of melanin [7]. While melanin generally functions as a protective pigment by absorbing and dissipating UV, recent findings suggest that melanin can also mediate DNA damage following UV exposure. After UV exposure, reactive oxygen species (ROS) are generated, which degrade melanin into high-energy monomers capable of transferring energy to DNA. This process can also result in the formation of CPDs even hours after UV exposure, a phenomenon termed “dark CPD formation”. These post-exposure CPDs are similar in structure to those formed by direct UVB exposure, further increasing the mutation burden in keratinocytes [9]. The generation of ROS and other reactive molecules, such as nitric oxide, exacerbates the DNA damage, leading to single strand breaks and oxidative lesions, contributing to the carcinogenic process in cSCC [10].

Despite the fact that the current literature has not provided a direct link between high cholesterol and the skin cancer risk [11], the role of cholesterol in cancer development has been acknowledged by numerous researchers [12]. Cholesterol crystallization in normal cells has been implicated in the onset of malignancy and altered blood cholesterol levels are frequently observed in cancer patients [12]. More recent findings suggest that cholesterol may act not only as a risk factor but also as a prognostic marker in certain cancers, with high-density lipoprotein (HDL) cholesterol influencing both patient survival and recurrence [13,14]. Cholesterol accumulation within tumors is often attributed to upregulated biosynthesis or increased uptake by cancer cells, many of which overexpress low-density lipoprotein receptors (LDLRs) to boost cholesterol intake and fuel rapid cell proliferation [15,16]. While the precise role of cholesterol in carcinogenesis remains a subject of debate, its involvement in tumor growth and recurrence highlights the need for further exploration of its role in cancer biology.

In the realm of cutaneous squamous cell carcinoma (cSCC) research, murine models play a pivotal role in elucidating the pathogenesis, metastatic potential, and therapeutic responses of this prevalent skin cancer. Among these models, the cell line-derived xenograft (CDX) and syngeneic mouse models stand out for their contributions to our understanding of cSCC. Additionally, the use of chemical and UV-induced carcinogenesis models further enriches the landscape of cSCC research [17]. The two-stage carcinogenesis model, employing agents like DMBA followed by TPA, has delineated the stages of tumor formation, highlighting the role of genetic mutations prevalent in cSCC [18].

UV-induced models, especially using hairless mice, mimic chronic sun exposure’s effects, providing insights into the role of UV radiation in cSCC’s pathogenesis and the specific genetic mechanisms involved [19]. These models offer significant advantages, including the ability to study the effects of carcinogens and UV radiation, despite also having limitations, such as not fully replicating the human condition, particularly in the case of metastatic disease [19,20].

To deepen our understanding of cSCC carcinogenesis, particularly under the influence of factors such as the melanin content, hypercholesterolemia, and immunosuppression, we conducted a comparative analysis using four distinct hairless mouse models exposed to UV light. Our main objective was to identify the most appropriate hairless animal model for cSCC experimental induction. By focusing on the melanin content and cholesterol level, this research aims to establish a foundation for future investigations of cSCC.

## 2. Materials and Methods

### 2.1. Animals

All the animal care procedures were conducted in strict adherence to the ARRIVE guidelines and those established by the European Council Directive. The experimental protocol received approval from the National Peripheral Veterinary Authority Animal Ethics Committee (Protocol Number 822470/12-12-2019). A cohort of 39 hairless male mice in four distinct strain groups was utilized in this study: 10 SKH-hr1, 10 SKH-hr2, 10 SKH-hr2+ApoE, and 9 Nude mice. The sample size was calculated using G*Power 3.1 [21], with a Cohen’s f of 0.8 to reflect a large effect size, assuming minimal within-group variability due to the use of genetically identical mice within each group. Based on this, a total of 9–10 animals per group was deemed sufficient to ensure adequate statistical power (80%) for detecting significant differences.

The Nude mice reported are FOXN1 “knockout” mice with an absent thymus gland. Consequently, they are partially immunosuppressed due to a significantly reduced number of T-cells. Additionally, they are albinos [22]. The SKH-hr1 mice are also albino and hairless, derived from BALB/c strains. They carry the hr/hr gene [23]. The SKH-hr2 mice are hairless mice with melanin, originating from the C57BL/6J strains. These mice also possess the hr/hr gene [24]. The SKH-hr2+ApoE mice are hairless with high cholesterol levels, lacking the expression of apolipoprotein E; hence, they are apolipoprotein E-deficient. They also secrete melanin. These mice inherently exhibit high total cholesterol with elevated LDL levels. This strain was developed in our laboratory following the crossbreeding of SKH-hr2 mice with ApoE mice [25] under the previously reported approval (Protocol Number 4044/14-07-2017).

All the animals were sourced from the breeding facilities of the School of Pharmacy Small Animal Laboratory (EL 25 BIO-Br 06). Environmental conditions within the animal housing facility were regulated to maintain a temperature of 24 ± 1 °C and humidity levels between 30 and 55%. Lighting was provided by yellow, fluorescent tubes, arranged to simulate a 12 h light/dark cycle (activated at 08:00 and deactivated at 20:00), emitting no detectable UV radiation. The mice had continuous access to solid food (Nuevo SA, Evoia—Farma Efyra, Korinthos, Greece) and tap water.

All the experimental animals were monitored throughout the study, with regular clinical evaluation of their general state of health, weight and temperature measurements to assess their overall health. Given that cSCC can present metastasis, albeit at low rates, necropsies were performed on all the animals at the conclusion of the experiment, with a thorough examination of their major organs. No significant weight loss or temperature increase was observed in any of the animals, nor was there any evidence of metastasis in any of them.

### 2.2. UV Irradiation

Solar-simulating UV radiation was administered using a 1000 W Xenon lamp positioned in an Arc Lamp Housing (66021), connected to a Universal Power Supply (68820) provided by Oriel Instruments (Irvine, CA, USA). The lamp setup included cooling and power supply adjustments, with the irradiation levels set at 9.5 mW/cm^2^ for UVA and 12 mW/cm^2^ for UVB. Daily pre-irradiation checks of the radiation dose were performed using a Coldilux Smart Meter (70239). The lower dorsal region of the animals was exposed to UV light three times a week for 32 weeks, starting with 1 Minimal Erythema Dose (MED) (57 mJ/cm^2^ for UVA and 72 mJ/cm^2^ for UVB) in the first week and incrementally increasing by 0.5 MED each subsequent week until reaching the stable dose of 3 MED (171 mJ/cm^2^ for UVA and 216 mJ/cm^2^ for UVB).

### 2.3. Clinical Evaluation—Photodocumentation

The progression of skin lesions to squamous cell carcinoma and the overall health of the mice were monitored five times per week, with the body weight measured once per month. Photodocumentation was performed weekly using a Nikon D5100 digital camera equipped with an AF-S Micro Nikkor 60 mm f/2.8 G ED lens (Nikon, Tokyo, Japan). Pre-cancerous symptoms, such as the occurrence of actinic keratosis and the appearance and number of papillomas, were evaluated. The time of onset, number per mouse, and size of squamous cell carcinomas were also assessed.

### 2.4. Measurements

Throughout the investigation, skin parameters were assessed using non-invasive biophysical techniques under controlled laboratory conditions. The transepidermal water loss (TEWL) was measured using a Tewameter TM 210 (Courage and Khazaka, Köln, Germany) to evaluate the skin’s water barrier function [26]. The skin hydration levels were determined via a Corneometer CM 820 (Courage and Khazaka, Köln, Germany), while the sebum production was quantified with a Sebumeter (Courage and Khazaka, Köln, Germany) [27]. The erythema and melanin concentrations were analyzed using a Mexameter MX 18 (Courage and Khazaka, Köln, Germany), which calculates the levels based on absorption differences at specific wavelengths [28]. The skinfold thickness was measured with a digital pachymeter by Casio.

### 2.5. Histological Analysis

Upon the study’s completion, the animals were sacrificed by cervical dislocation. Skin samples were harvested from each group and preserved in formalin for histopathological examination conducted at the Athens Naval Hospital’s histopathology laboratory. Serial 5 μm tissue sections were obtained, deparaffinized, and stained with hematoxylin–eosin (HE) stain. The skin tissue slides were examined and evaluated randomly under blinded conditions. The evaluation focused on the extent and morphological characteristics of squamous cell carcinoma.

Color images were captured using an Olympus BX43 light microscope equipped with a digital camera (Olympus Life Sciences, Center Valley, PA, USA).

### 2.6. Proteasome Activity Assay

Fresh tissue (normal skin or tumor) was flash frozen in liquid nitrogen (LN2) and pulverized with the help of a porcelain pestle while immerged in LN2. Small amounts of pulverized tissue were transferred into tubes, where they were resuspended in lysis buffer and sonicated three times for 30 s (with 30 s pauses) in a frozen water bath. The lysis buffer contained 20 mM Tris/HCl lysis buffer, pH 7.6, 5 mM ATP, 10% glycerol, 20 mM KCL, 1 mM EDTA, 1 mM DTT, 0,2% Nonidet P-40, 1 mM phenylmethylsulfonyl-fluoride and 10 μg/mL aprotinin [17]. The lysates were centrifuged at 13,000× *g* rpm for 10 min and the supernatants were used to determine the proteasome activities. The chymotrypsin-like (CT-L), trypsin-like (T-L) and caspase-like (C-L) proteasome activities were determined in duplicates or triplicates after incubating 30 μg of total protein for 1 h at 37 °C with the Suc-LLVY-AMC, Boc-LRR-AMC and Z-LLE-AMC fluorogenic peptides (UBPBio, Aurora, CO, USA) for the determination of CT-L, T-L and C-L activities, respectively, as previously described [29]. Proteasomal activity was determined as the difference between the total activity and the activity in the presence of 20 μΜ MG132 (proteasome inhibitor). In all cases, methyl coumarin (MCA) liberation was measured every 5 min using the Safire2 Multi-Detection Microplate Reader (Tecan, Grodig, Austria) at λex = 380 nm, λem = 460 nm at 37 °C for 30 min. The protein concentration was determined by Bradford assay using bovine serum albumin (BSA) as standard. Proteasome activity was determined as the fold-change (%) of the relative specific proteasome activity in normal (N) tissues that was set to 1.

### 2.7. Immunoblot Analysis

First, 20 μg of total protein extracted in the lysis buffer described above was separated by 12% SDS-PAGE. The proteins were then transferred to nitrocellulose membranes for probing with appropriate antibodies. Secondary antibodies conjugated with horseradish peroxidase (anti-mouse, sc-516102, Santa Cruz, TX, USA) were used to detect the bound primary antibodies (anti-β1, BML-PW8140, Enzo Life Sciences, New York, NY, USA, anti-β2, BML-PW814, Enzo Life Sciences). All the blots were developed with chemiluminescence using the Clarity^TM^ Western ECL substrate (Bio-Rad Laboratories, Hercules, CA, USA) in a ChemiDoc station (Bio-Rad Laboratories, Hercules, CA, USA). Coomassie Brilliant Blue staining was used to check the equal loading of the protein samples. Densitometry analysis for the quantification of the blots was performed with ImageJ (version 1.53f, 25 October 2020). The molecular weight of each protein appears on the right of each blot.

### 2.8. Sebum Levels

The sebum levels within the lower back region were quantified utilizing the Sebumeter^®^ device from Courage-Khazaka Electronic GmbH, Köln, Germany, which was connected to an MPA base unit. The apparatus employs a cassette featuring a measurement section equipped with a tape that contacts the skin for a duration of 30 s. Subsequently, the cassette is placed into a designated slot, where a light source assesses the transparency of the tape. This assessment operates on the grease-spot photometry principle, with a microprocessor performing the calculations to determine the sebum levels, presenting the findings in mg of sebum per cm^2^ of skin surface area [30]. The measurements were manually logged for each participant, with the specific cheekbone side (right or left) chosen at random for testing.

### 2.9. Statistical Methodology

Data analysis was performed using GraphPad Prism 8 software. The results were presented as the mean value ± SD unless otherwise indicated. All the collected data underwent a normality test using the Shapiro–Wilk test. Depending on the normality of the distribution, statistical significance was determined through adequate analysis (paired or unpaired *t*-test, one-way ANOVA, post hoc analysis LSD or non-parametric Wilcoxon test), with a 95% confidence level and significance threshold set at *p* < 0.05 (depicted as *). Part of the data analysis was conducted using Jamovi software (version 2.3, 2022), including binomial logistic regression, and generalized linear models to assess the incidence, timing, and count of the papilloma and tumor formation across different animal models [31].

## 3. Results

### 3.1. Clinical Evaluation

#### 3.1.1. Photodocumentation

**Figure 1:**
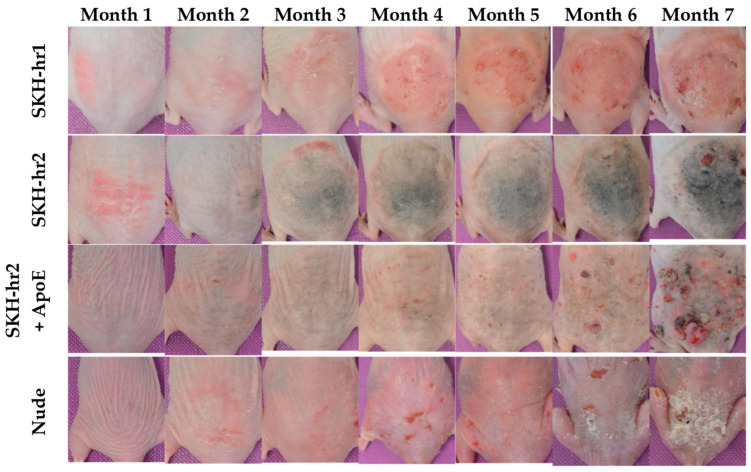
Evolution of carcinogenesis over time. Representative images of the four irradiated mouse models (SKH-hr1, SKH-hr2, SKH-hr2+ApoE, and Nude) at various time points. Differences are evident as early as the first month across all the groups, with increased melanin observed in the skin of the SKH-hr2 and SKH-hr2+ApoE models by the third month. Papilloma formation began after the third month, and tumor development was noted after the seventh month in the SKH-hr2, SKH-hr2+ApoE, and Nude models. In contrast, the SKH-hr1 mice primarily exhibited actinic keratosis toward the study’s end, with minimal papilloma and tumor formation.

Τhe skin of all the mice exhibited signs of erythema, burns, scaling, and hyperkeratosis right from the onset of radiation exposure (Figure 1). The SKH-hr2 and SKH-hr2+ApoE models began to secrete melanin within the initial weeks of radiation.

Significant differences were noted in the clinical conditions among the four mouse models (Figure 1). The SKH-hr1 model’s irradiated skin gradually thickened from the third month onwards due to hyperkeratosis, developing actinic keratosis (AK) by the fourth month, which did not progress to skin cancer until the conclusion of the study. A few SCCs, appearing as small wounds and tumors, were noted in the eighth month. Within the SKH-hr1 mice, which predominantly developed actinic keratosis, a precancerous condition, only a minority developed papillomas and SCCs as small open wounds or tumors.

The Nude mouse model exhibited dryness and exfoliation from the sixth month, with the first SCCs appearing by the seventh month. The eighth month saw numerous small SCCs, along with intense exfoliation. Additionally, two mice developed colorectal cancer, which likely led to their demise. Moreover, the Nude mice exhibited severe skin scaling and SCCs as elevated tumors and wounds with crusts (Figure 1).

By the sixth month, the SKH-hr2 model showed the development of papillomas and wounds of varying sizes. Tumors were observed in some mice by the seventh month. Most of the SKH-hr2 mice developed advanced, invasive SCCs, manifesting as craters, non-healing wounds that occasionally bled, as well as elevated, solid tumors of substantial size with crusty surfaces. These tumors exhibited severe internal circulation but no signs of metastasis.

The SKH-hr2+ApoE model presented with small wounds by the fourth month, and the emergence of the first papillomas was noted by the fifth month. There was a noticeable increase in the number of papillomas during the sixth and seventh months, with the first SCCs appearing by the seventh month. Most SKH-hr2+ApoE mice developed SCCs, some originating from multiple papillomas that grew and merged into large, rough masses. In certain instances, the carcinomas presented as elevated tumors with red bases, crusty surfaces, and circulation, or as open, non-healing wounds. A few mice also showed the tissue under the tumor degenerating, indicating tumor infiltration into surrounding tissues.

#### 3.1.2. Number of Papillomas and Tumors Formation over Time

**Figure 2:**
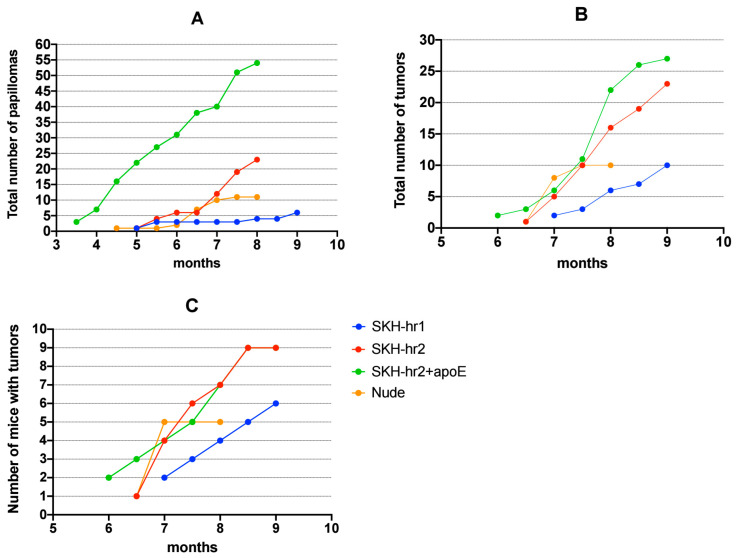
Cumulative counts of papillomas and tumors over time. (**A**) Cumulative number of papillomas per mouse strain. (**B**) Cumulative number of tumors per mouse strain. (**C**) Cumulative number of animals exhibiting carcinogenesis per mouse strain (*n* = 10 SKH-hr1, 10 SKH-hr2, 10 SKH-hr2+ApoE, and 9 Nude mice).

The SKH-hr2+ApoE model developed its initial papillomas in the third month, the Nude model in the fourth month, and both the SKH-hr1 and SKH-hr2 models in the fifth month. Compared to the other three models, the SKH-hr2+ApoE model exhibited a higher papilloma count, with the SKH-hr1 model developing the fewest. Figure 2A displays the monthly count of papillomas for each mouse model.

Figure 2B depicts the monthly tumor count across the mouse models. The SKH-hr2+ApoE model manifested the first SCCs in the sixth month, preceding the SKH-hr2 and Nude models, which developed SCCs later in the same month, and the SKH-hr1 model in the seventh month. The SKH-hr2+ApoE and SKH-hr2 models exhibited a higher SCC count compared to the Nude and SKH-hr1 models. Figure 2C shows that a notably high percentage of the SKH-hr2+ApoE and SKH-hr2 mice developed cSCC up to experiment’s conclusion.

The descriptive statistics presented in Table 1 illustrate the cumulative number of papillomas and tumors across the different mouse models, along with their respective 95% confidence intervals (CI) and Shapiro–Wilk test results for normality. The SKH-hr2+ApoE model exhibited the highest mean number of papillomas (M = 3.233, 95% CI [2.3882, 4.078]), significantly greater than the other models, followed by the SKH-hr2 (M = 1.042) and Nude (M = 0.698) models, with the SKH-hr1 model showing the lowest mean (M = 0.175). In terms of tumor formation, the SKH-hr2+ApoE model again showed the highest mean (M = 0.850), while the SKH-hr1 model had the lowest mean (M = 0.183).

Based on Table 2 and Table 3, it appears that the formation of papillomas is more likely, both in terms of the probability of occurrence (Table 2) and the severity of the phenomenon (Table 3), in species that have melanin, particularly in the Skh-hr2 + ApoE model. However, a similar pattern is observed in the Nude model, although with lower probability and intensity. The Skh-hr1 model does not seem to differ significantly in papilloma formation compared to the non-irradiated animals, which we have frequently observed in the past to have an almost negligible probability of developing papillomas.

The results regarding the probability and intensity of squamous cell carcinoma formation show a similar pattern to the papilloma findings (Table 4 and Table 5). However, in this case, the probability of tumor formation was calculated to be similar in the Nude, Skh-hr2, and Skh-hr2 + ApoE models (Table 4). The Skh-hr1 model does not appear to differ significantly from the non-irradiated animals, at least in terms of the carcinogenesis probability. However, the intensity of the phenomenon was marginally non-significant. Additionally, according to Table 5, the odds ratios concerning the intensity of carcinogenesis suggest a potentially increased tendency in melanin-bearing animals compared to the Nude model, although this difference is much smaller than the differences observed in papilloma formation. Once again, the Skh-hr2 + ApoE model was found to have the highest odds ratio.

### 3.2. Histopathological Evaluation

**Figure 3:**
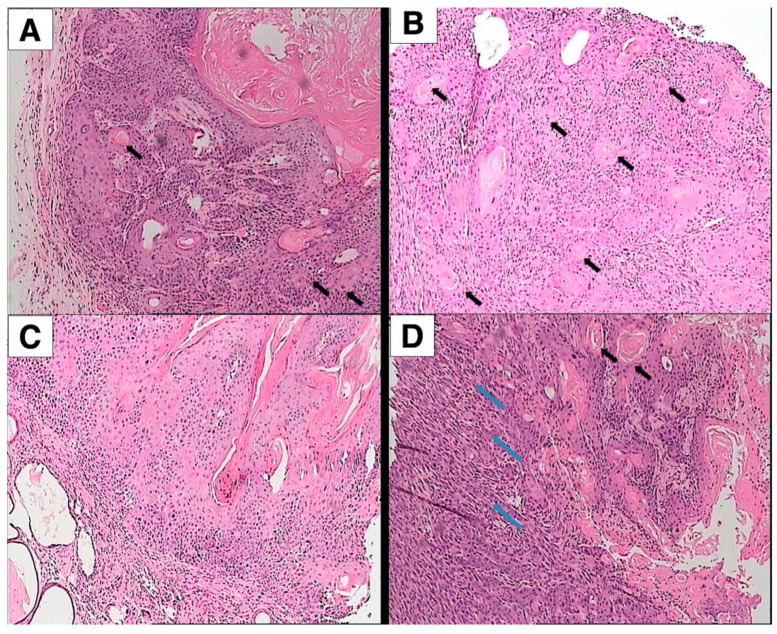
Histopathological assessment of squamous cell carcinoma. (**A**) SCC exhibiting numerous keratin pearls (indicated by black arrows), extending from the epidermis into the dermis. (**B**) Aggressively, poorly differentiated SCC characterized by the presence of abundant keratin pearls (marked by black arrows). (**C**) Actinic keratosis is noted, alongside an evolving SCC in situ that remains non-invasive and has yet to breach the basement membrane. (**D**) Aggressive SCC displaying significant mitotic activity and the presence of enlarged, atypical nuclei. To the right of the image, a well-differentiated region of the SCC features numerous keratin pearls (black arrows), in contrast to the left side, which shows a poorly differentiated region of the SCC with cohesive cell clusters (blue arrows). Panels (**A**–**D**) correspond to Nude and SKH-hr2+ApoE, SKH-hr1, and SKH-hr2 mice, respectively. (Magnification: ×100).

Figure 3 presents the histopathological analysis of representative skin samples from each type of mouse after sacrifice at the end of the experiment. The SCCs were characterized by large, irregular masses of epidermal cells with enlarged, atypical nuclei undergoing frequent mitosis. The squamous cells varied significantly in size and shape, exhibiting hyperplasia, hyperchromasia of nuclei, and atypical mitotic figures. Keratinization, indicative of squamous cell carcinoma differentiation, occurred in the form of horn pearls, distinctive structures comprising layers of squamous cells with increasing keratinization toward the center. Most SCCs in the SKH-hr2, SKH-hr2+ApoE, and Nude models were invasive and ranged from moderately to poorly differentiated, often containing horn pearls indicative of well- or moderately differentiated SCCs. The SKH-hr1 mice showed fewer SCCs but a higher incidence of actinic keratosis, with occasional development of in situ SCCs, which had not yet become invasive.

### 3.3. Biophysical Evaluation

**Figure 4:**
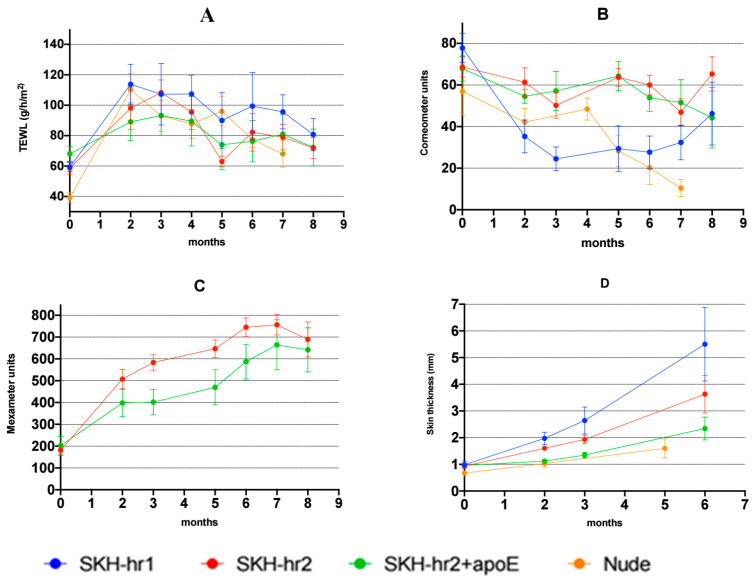
Graphical representation of the alterations in the skin parameters per month. (**A**) Transepidermal water loss. (**B**) Hydration. (**C**) Melanin. (**D**) Skin thickness. Each mouse model is depicted with a different color (blue for SKH-hr1, red for SKH-hr2, green for SKH-hr2+ApoE, orange for Nude).

The variations in the transepidermal water loss (TEWL) values for each mouse model throughout the experiment are illustrated in Figure 4A. The transepidermal water loss (TEWL) was measured as an indicator of skin barrier function, providing insights into the skin condition, including the intensity of inflammation and potential structural changes. A significant rise in TEWL was noted for all the models two months post-UV radiation. Following this initial increase, the TEWL slightly declined over the next three months but never returned to the pre-radiation level for any model.

Figure 4B outlines the changes in skin hydration across the mouse models during the experiment. Two months post-UV radiation, a significant reduction in hydration was observed for all the models, particularly in the SKH-hr2. This decrease persisted into the third month for the SKH-hr1 and SKH-hr2, while the SKH-hr2+ApoE and Nude models saw a non-statistically significant rise in skin hydration between the second and fourth months. Post the fifth month, hydration decreased for all the models except for the SKH-hr1, which exhibited an increase in hydration until the study’s end. Additionally, the epidermal hydration of the SKH-hr2 model increased in the eighth month of radiation (*p* < 0.05).

The melanin level changes in the epidermis of the SKH-hr2 and SKH-hr2+ApoE models are presented in Figure 4C. The SKH-hr1 and Nude models were excluded from this graph as their skin did not produce melanin. The initial melanin levels between the SKH-hr2 and SKH-hr2+ApoE models showed no statistically significant difference (*p* = 0.16). A statistically significant increase in the melanin values for both models was observed in the subsequent months (*p* < 0.05), with the SKH-hr2 model consistently displaying higher melanin levels. The final melanin levels between the SKH-hr2 and SKH-hr2+ApoE models were not significantly different (*p* = 0.28).

The skinfold thickness results for each mouse model can be found in Figure 4D. The initial skinfold thickness values between the SKH-hr2, SKH-hr1, and SKH-hr2+ApoE models showed no statistically significant differences (*p* > 0.05), but the skinfold thickness of the Nude mice was significantly lower than that of the other models (*p* < 0.05). The skinfold thickness significantly increased for all the models, with the greatest increase observed in the SKH-hr1 model and the least in the Nude model.

### 3.4. Proteasome Activities and Expressions

**Figure 5:**
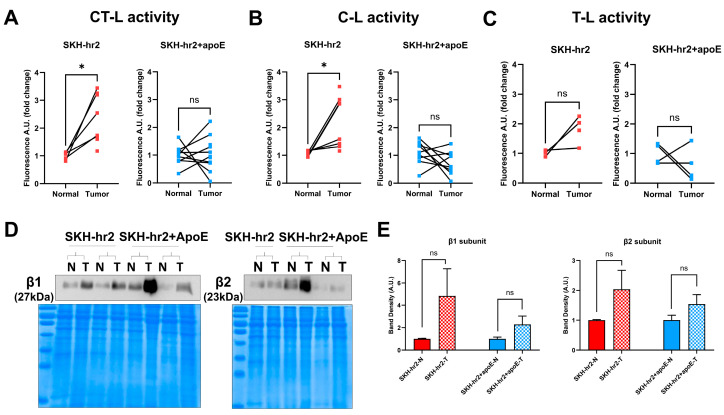
Proteasome status in the SKH-hr2 and SKH-hr2+ApoE mice: (**A**) chymotrypsin-like (CT-L), (**B**) caspase-like (C-L) and (**C**) trypsin-like (T-L) activities measured in normal (N) and tumorous (T) skin of hr2 (left) and hr2-ApoE (right) animals. Tissues from (**A**) 5 (N) -8 (T) hr2 animals and 9 (N) -11 (T) hr2-ApoE animals, (**B**) 6 (N) -8 (T) hr2 animals and 9 (N and T) hr2-ApoE animals, and (**C**) 3 (N) -4 (T) hr2 animals and 4 (N and T) hr2-ApoE animals were analyzed (paired *t*-test for (**A**,**B**), Wilcoxon test for (**C**), * *p* value < 0.05, ns, non-significant). (**D**) Representative immunoblots and (**E**) Band densitometry for the β1 and β2 proteasome subunits, depicting the mean band density ± SEM. The braces indicate that the normal (N) and tumorous (T) tissue came from the same animal. The β1 and β2 protein levels were normalized to gels stained with Coomassie Brilliant Blue. Tissues from 5–8 animals per group were analyzed (unpaired *t*-test, ns, non-significant). The original Western blot figures can be found in Supplemental Materials.

The proteasome function and expression have been shown to be affected during UV exposure [32], while the enhanced sensitivity of cSCC cell lines to proteasome inhibitors has been shown before [33], thus suggesting a possible role of proteasome in cutaneous squamous cell carcinogenesis. Therefore, we assessed the three proteasome activities in normal and tumorous skin from the SKH-hr2 and SKH-hr2+ApoE animals. The CT-L and C-L activities were found to be significantly enhanced in tumors from the SKH-hr2 animals as compared to normal skin coming from the same animals (Figure 5A,B; SKH-hr2, normal (N) compared to tumors (T)). A similar tendency was shown for the T-L activity without, however, reaching statistical significance, probably due to the low sample number (Figure 5C; SKH-hr2). In contrast, no differences were observed between normal and tumorous tissues from the SKH-hr2+ApoE animals (Figure 5A–C; SKH-hr2+ApoE, normal (N) compared to tumors (T)). This could suggest a pivotal role of the ApoE protein in the regulation of proteasome activity in tumors; ApoE deficiency may not allow the enhancement of proteasome activities in the skin tumors of these animals.

We then evaluated the protein expression levels of the β1 (catalytic center of C-L activity) and β2 (catalytic center of T-L activity) proteasome subunits to check whether the enhanced levels of proteasome activities are related to enhanced protein expression of the proteasome subunits (Figure 5E). Although there is a trend of upregulation for both subunits in the tumors in both SKH-hr2 and SKH-hr2+ApoE animals (with the trend being less obvious in the SKH-hr2+ApoE animals, thus potentially attributing a regulatory role to ApoE protein), none of them reached statistical significance (Figure 5E). Consequently, the observed enhanced proteasome activities seem to be regulated at the functional level (activity) and not the expression level. In total, the observed enhancement of the proteasome activities in these tumors may be linked to the heightened requirement for the degradation of proteins associated with rapid cellular proliferation, the removal of misfolded proteins produced under tumorigenic stress, or the circumvention of apoptosis by cancerous cells.

### 3.5. Sebum Level Measurements

**Figure 6:**
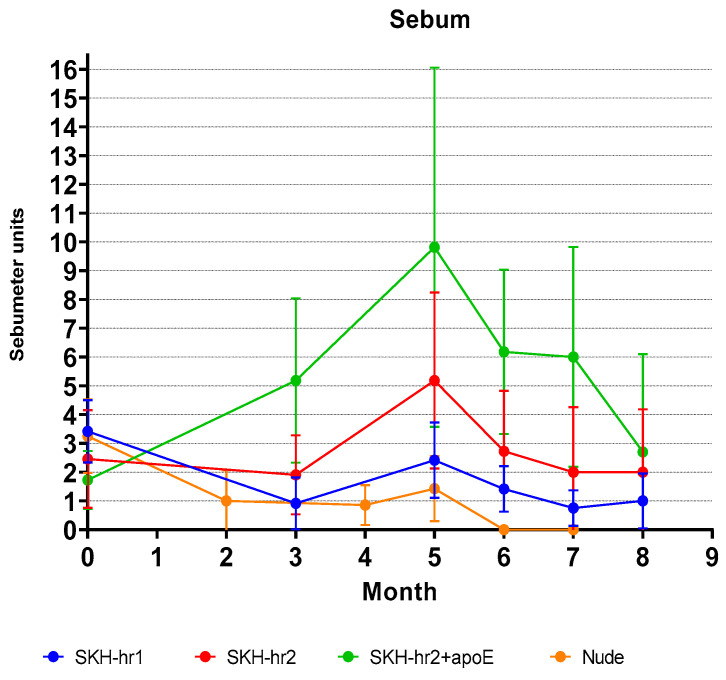
Sebum measurements across different murine models over time. While no statistically significant differences in the skin sebum levels were detected across the groups, the SKH-hr2+ApoE mice consistently exhibited higher average sebum concentrations compared to the other models.

The sebum levels on the skin surface were quantified. Sebum’s primary components are ceramides, triglycerides, squalene, and cholesterol. Given its susceptibility to UV-induced oxidation, sebum quantification may be related to skin oxidative stress. In Figure 6, the mean values and standard deviations (SDs) of the sebum measurements for each month of the experiment are presented. The measurements do not follow a normal distribution, and the Wilcoxon test does not show any statistically significant differences between the mice (*p* > 0.05). However, the SKH-hr2+ApoE mice demonstrated a greater increase in the sebum levels, which may be associated with their hypercholesterolemia, as previously suggested [21].

## 4. Discussion

The observed clinical manifestations of squamous cell carcinoma in the SKH-hr2+ApoE and SKH-hr2 murine models present significant insights into the complexity of skin cancer development under the influence of genetic and UV radiation factors. The earlier onset of papillomas and cancer in the SKH-hr2+ApoE models, followed by the later appearance but rapid growth of cSCC in the SKH-hr2 models (Figure 1 and Figure 3), underscores a complex interplay between genetic predispositions and the carcinogenic process. Notably, the absence of melanoma in both models, despite the aggressive nature of the SCCs observed (Figure 2), suggests a specific pathway of carcinogenesis that is potentiated by UV exposure yet distinct in its etiology and progression compared to melanoma, as already reported [34]. The relatively limited extent of the SCCs observed in the SKH-hr1 mice compared to the SKH-hr2+ApoE, SKH-hr2 and Nude mice, alongside the low rate of precancerous conditions such as actinic keratosis in the SKH-hr1 mice, further illustrates the variable susceptibility and response of different genetic backgrounds to carcinogenic stimuli. This ability of the SKH-hr1 mice in association with the much higher skin thickness induced (Figure 5D) could at least partly explain the strain’s resistance to the development of SCC despite the genetic absence of melanin.

The common characteristic of the SKH-hr2+ApoE and SKH-hr2 murine models is melanin. Melanin is known as an efficient filter. Based on the findings presented in this study (Table 2 and Table 3), the Skh-hr2 and Skh-hr2+ApoE models appear to be particularly valuable for studying precancerous papillomas. This is especially true for the Skh-hr2+ApoE model, where the formation of papillomas occurs in significant numbers and relatively early (Figure 2 and Table 3).

The increase evidenced in the transepidermal water loss across all the mouse types due to UV radiation-induced epidermal barrier disruption (Figure 5A) highlights the pivotal role of the skin’s barrier function in maintaining hydration and homeostasis. Interestingly, despite the universal decrease in hydration across all the mouse types, it was the Nude mice that displayed the most significant reduction (Figure 5B), pointing toward a potentially heightened sensitivity to UV radiation-induced damage in these mice. This sensitivity could be attributed to the immunosuppression and absence of protective hair or pigmentation, which in other mouse types might mitigate the effects of UV exposure to some degree [35]. The specific increase in erythema in the SKH-hr1 mice (Figure 5C) from the onset of radiation exposure further suggests a differential susceptibility to UV radiation among the mouse types, possibly due to variations in skin structure, the immune response through mast cells, or the inability to induce protective responses such as melanin production, as reported elsewhere [36]. Skin thickness due to hyperkeratosis as a direct result of UV exposure (Figure 5D), most notably in the SKH-hr1 mice, seems to also be a significant factor in squamous cell carcinogenesis. The production of melanin in both the SKH-hr2 and SKH-hr2+ApoE mice (Figure 5C), with the highest increases and levels detected in SKH-hr2, indicates a protective adaptation against UV radiation, absent in the albino SKH-hr1 and Nude mice, which controversially may enhance photosensitization through pro-oxidant effects [37].

The histopathological evaluation revealing aggressive, poorly differentiated SCC in both the SKH-hr2 and SKH-hr2+ApoE models, in contrast to the less aggressive SCC found in the Nude mice, and the mix of actinic keratosis, SCC in situ, and invasive SCC in the SKH-hr1 models (Figure 4), provides a microscopic confirmation of the clinical observations. This differentiation in the histopathological characteristics of SCC among the mouse models may reflect differences in the molecular mechanisms underlying tumor progression. The presence of more aggressive and less differentiated tumors in the SKH-hr2 and SKH-hr2+ApoE mice suggests a more pronounced breakdown in cellular control mechanisms, potentially implicating the role of apolipoprotein E in modulating the tumor microenvironment or influencing the behavior of cancer cells, as also supported by previous findings [38,39].

Given the upregulation of the proteasome activities in the tumors of the SKH-hr2 but not the SKH-hr2-ApoE mice, the hypothesis that apolipoprotein E is necessary for the activation of the proteasome in tumors gains traction. This posits that while apolipoprotein E may not directly affect the expression of the catalytic subunits of the proteasome, it could play a crucial role in the assembly or activation of the proteasome complex within the tumor microenvironment. Such a role for apolipoprotein E could be pivotal in understanding the degradation pathways that are enhanced or inhibited in the context of tumorigenesis, offering insights into the mechanisms through which apolipoprotein E modulates the proteasome function and influences the progression of cancer.

The phenomenon, namely the upregulation of proteasome activities in the tumors of SKH-hr2 mice, contrasted with the lack of such upregulation in the SKH-hr2-ApoE mice, provides an additional perspective on the value of the Skh-hr2+ApoE mouse model, as this model may be valuable for studying therapeutic agents that inhibit the proteasome in mammals.

The SKH-hr2+ApoE mice demonstrated an increase in the sebum levels (Figure 6), which could be associated with the strain genetic hypercholesterolemia. Increased lipid levels in the epidermis following UV radiation exposure have been proven to elevate the levels of 4-hydroxy-2-nonenal (HNE) as a byproduct of lipid peroxidation [40]. HNE, in turn, has been identified in high concentrations in cases of squamous cell carcinoma [41]. Thus, the findings of the present study prompt further inquiry into the etiopathological relationship between increased sebum and the development of squamous cell carcinoma after UV irradiation. This suggests that HNE may play a critical role in the signaling pathways that regulate tumor growth, particularly through its interaction with the proteins in the tumor and its surrounding non-tumorous tissue.

The present study, despite its significant findings, possesses certain limitations, which include the absence of a complete mechanistic approach that would contribute to the understanding of the carcinogenic processes. It is true that our mechanistic approach is limited, as only the proteasome activity in relation to skin cancer development was studied. It is an important future task to elucidate the specific mechanisms of carcinogenesis in this context. In particular, following the observation that the carcinogenesis achieved may be more dependent on secondary DNA damage mediated by melanin, a more solid explanation for the mechanism of carcinogenesis shall be provided in the future. Moreover, further studies should investigate the role of apolipoprotein E deficiency in contributing to carcinogenesis, as indicated by this study’s findings. Addressing these mechanistic questions would provide a more comprehensive understanding of cSCC development.

Nevertheless, despite these limitations, the following results are clearly delivered. Initially, the murine models SKH-hr2+ApoE and SKH-hr2 were identified as models for the generation of cSCC to be used in future experiments, and notably, with significant advantages compared to the Nude (FOXN1 knockout) and SKH-hr1 mice currently mentioned in the literature [30].

The question of whether the observed differences in the relative SKH-hr2 and SKH-hr2+ApoE mice—such as the number of papillomas, the timing of squamous cell carcinoma (SCC) and papilloma appearance, and the CT-L and C-L proteasome activities—could be attributed to the deficiency of apolipoprotein E and/or the consequent hyperlipidemia exhibited by the SKH-hr2+ApoE mice is a rational one.

The common characteristic of the SKH-hr2+ApoE and SKH-hr2 murine models is melanin. Melanin is known as an efficient filter against UV light possessing antioxidant capacity [35]. However, the presence of metal ions like iron and copper melanin could act as a pro-oxidant [42]. It is known that in the presence of UV-induced inflammation, the iron concentration in cells is enhanced [43]. Moreover, it has previously been proposed that melanin can act as both a carcinogenic agent and a protective factor against cancer. This dual behavior is particularly observed following UVA exposure, where UV-induced reactive oxygen and nitrogen species interact with melanin fragments. This results in a quantum triplet state that holds the energy equivalent to a UV photon that, in turn, induces CPD formation by energy transfer to DNA in a radiation-independent manner [42]. The possibility, after surpassing a dose of UV light, to obtain with the above strains similar phenomena seems interesting and will be investigated soon.

The use of SKH-hr2 and SKH-hr2+ApoE mice as models to investigation possible new therapeutic interventions and drugs and to the approach of their mechanism of action could significantly help the treatment of SCCs in the future.

## 5. Conclusions

The present study underscores the distinct differences in the cutaneous squamous cell cancer (cSCC) susceptibility and progression among the four murine models (SKH-hr1, SKH-hr2, SKH-hr2+ApoE, and Nude), with the Nude, SKH-hr2 and SKH-hr2+ApoE mice demonstrating a higher propensity for papilloma and tumor development, influenced by both genetic factors and UV radiation, while the SKH-hr2 and SKH-hr2+ApoE models seem to exhibit the phenomenon with greater intensity. The differential proteasome activity observed between these models highlights the potential role of apolipoprotein E in modulating tumorigenesis, offering valuable insights for future research into therapeutic targets for SCC. Additionally, the findings suggest that the SKH-hr2 and SKH-hr2+ApoE models are particularly well suited for investigating the pathophysiology of cSCC and testing novel therapeutic interventions, making them promising tools for advancing skin cancer treatment.

## Figures and Tables

**Table 1:** Descriptive statistics of cumulative tumor and papilloma counts, including the mean, 95% confidence intervals (CI), and standard deviation (SD) in each animal model. The Shapiro–Wilk test was used to assess the normality of the distribution for each group. The results indicate the central tendency and variability of papilloma and tumor formation across the different models, with all the groups showing significant deviation from distribution normality (*p* < 0.001).

			95% Confidence Interval			Shapiro–Wilk	
	Animal Model	Mean	Lower	Upper	SD	W	* p *
Number of papillomas	Skh-hr1	0.175	0.106	0.244	0.382	0.46	<0.001
	Skh-hr2	1.042	0.7955	1.288	1.362	0.736	<0.001
	Nude	0.698	0.4697	0.926	1.064	0.663	<0.001
	Skh-hr2+ApoE	3.233	2.3882	4.078	4.676	0.699	<0.001
Number of tumors	Skh-hr1	0.183	0.0572	0.309	0.698	0.288	<0.001
	Skh-hr2	0.65	0.4882	0.812	0.895	0.722	<0.001
	Nude	0.593	0.4273	0.759	0.773	0.711	<0.001
	Skh-hr2+ApoE	0.85	0.637	1.063	1.179	0.732	<0.001

Note: The CI of the mean assumes the sample means follow a t-distribution with N − 1 degrees of freedom.

**Table 2:** Binomial logistic regression model coefficients for the papilloma formation timepoint across the different animal models. The estimates represent the log odds of developing papillomas versus not developing papillomas as influenced by the time (in months) and the animal model. The intercept, Animal model (relative to the Skh-hr1 model), is shown with the corresponding standard error (SE), Z-value, and *p*-value. Significant predictors are highlighted, with *p*-values < 0.001 indicating a strong relationship between the predictor and the likelihood of papilloma formation. As seen, the Skh-hr1 group does not seem to differ from the baseline status (non-irradiated, considered to have no risk of developing papillomas).

Predictor	Estimate	SE	Z	*p*
Animal Model				
Skh-hr2 vs. Skh-hr1	1.812	0.3512	5.1594	<0.001
Nude vs. Skh-hr1	1.571	0.3795	4.1383	<0.001
Skh-hr2+ApoE vs. Skh-hr1	2.701	0.3684	7.331	<0.001
Non-irradiated vs. Skh-hr1	−18.003	866.143	−0.021	0.983

**Table 3:** Generalized linear model (Poisson regression) parameter estimates for the papilloma counts across the different animal models. The table includes the estimated coefficient (Estimate), standard error (SE), odds ratio, and the 95% confidence interval for the odds ratios (Lower and Upper). The z-values and *p*-values indicate the statistical significance of each model’s effect. Significant predictors, such as Skh-hr2, Nude, and Skh-hr2+ApoE relative to Skh-hr1, show strong associations with increased papilloma counts (*p* < 0.001), while the baseline comparison does not show a significant effect, suggesting that the Skh-hr1 group does not seem to differ from the baseline status (non-irradiated, considered to have no risk of developing papillomas).

						95% Exp(B) Confidence Interval	
Effect	Estimate	SE	Odds Ratios	Lower	Upper	z	* p *
Skh-hr2 vs. Skh-hr1	1.78	0.236	5.9524	3.84	9.72	7.5637	<0.001
Nude vs. Skh-hr1	1.38	0.254	3.9867	2.47	6.7	5.4545	<0.001
Skh-hr2+ApoE vs. Skh-hr1	2.92	0.224	18.4762	12.23	29.57	13.0174	<0.001
Non-irradiated vs. Skh-hr1	−16.56	521.937	6.43 × 10^−8^	0	0	−0.0317	0.975

**Table 4:** Binomial logistic regression model coefficients for squamous cell carcinoma formation for predicting the formation of squamous cell carcinoma (SCC) across the different animal models. The estimates represent the log odds of developing SCC versus not developing SCC as influenced by the time (in months) and the animal model. The table includes the estimated coefficient (Estimate), standard error (SE), Z-value, and *p*-value for the intercept, time, and each animal model relative to the Skh-hr1 model. Significant predictors (*p* < 0.001) indicate a strong association between the predictor variables and the likelihood of SCC formation.

Predictor	Estimate	SE	Z	*p*
Animal Model				
Skh-hr2 vs. Skh-hr1	4.51	0.649	6.9547	<0.001
Nude vs. Skh-hr1	5.8	0.773	7.4958	<0.001
Skh-hr2+ApoE vs. Skh-hr1	5.06	0.685	7.3883	<0.001
Non-irradiated vs. Skh-hr1	−18.2	1192.95	−0.0153	0.988

**Table 5:** Generalized linear model (Poisson regression) parameter estimates for tumor formation across the different animal models relative to the Skh-hr1 model. The table includes the estimated coefficient (Estimate), standard error (SE), odds ratio, and the 95% confidence interval for the odds ratios (Lower and Upper). The z-values and *p*-values indicate the statistical significance of each model’s effect. Significant positive effects were observed for the Skh-hr2, Nude, and Skh-hr2+ApoE models (*p* < 0.001), indicating a higher likelihood of tumor formation compared to the Skh-hr1 and the non-irradiated, considering that the comparison between the non-irradiated and Skh-hr1 was non-significant (*p* = 0.081).

						95% Exp(B) Confidence Interval	
Effect	Estimate	SE	Odds Ratios	Lower	Upper	z	* p *
Skh-hr2 vs. Skh-hr1	0.467	0.1047	1.595	1.299	1.96	4.46	<0.001
Nude vs. Skh-hr1	0.41	0.1146	1.506	1.203	1.89	3.57	<0.001
Skh-hr2+ApoE vs. Skh-hr1	0.667	0.1047	1.948	1.586	2.39	6.36	<0.001
Non-irradiated vs. Skh-hr1	−0.183	0.1047	0.832	0.678	1.02	−1.75	0.081

## Data Availability

The data presented in this study are available on request from the corresponding author.

## Supplementary Materials

The following supporting information can be downloaded at: https://www.mdpi.com/article/10.3390/cancers16203546/s1, The original Western blot figures.
