# Supplementary figures and images for: Comparative Study of Cutaneous Squamous Cell Carcinogenesis in Different Hairless Murine Models
Source: Cancers (Basel). 2024 Oct 21;16(20):3546. doi: 10.3390/cancers16203546 (PMC11506169; doi:10.3390/cancers16203546)

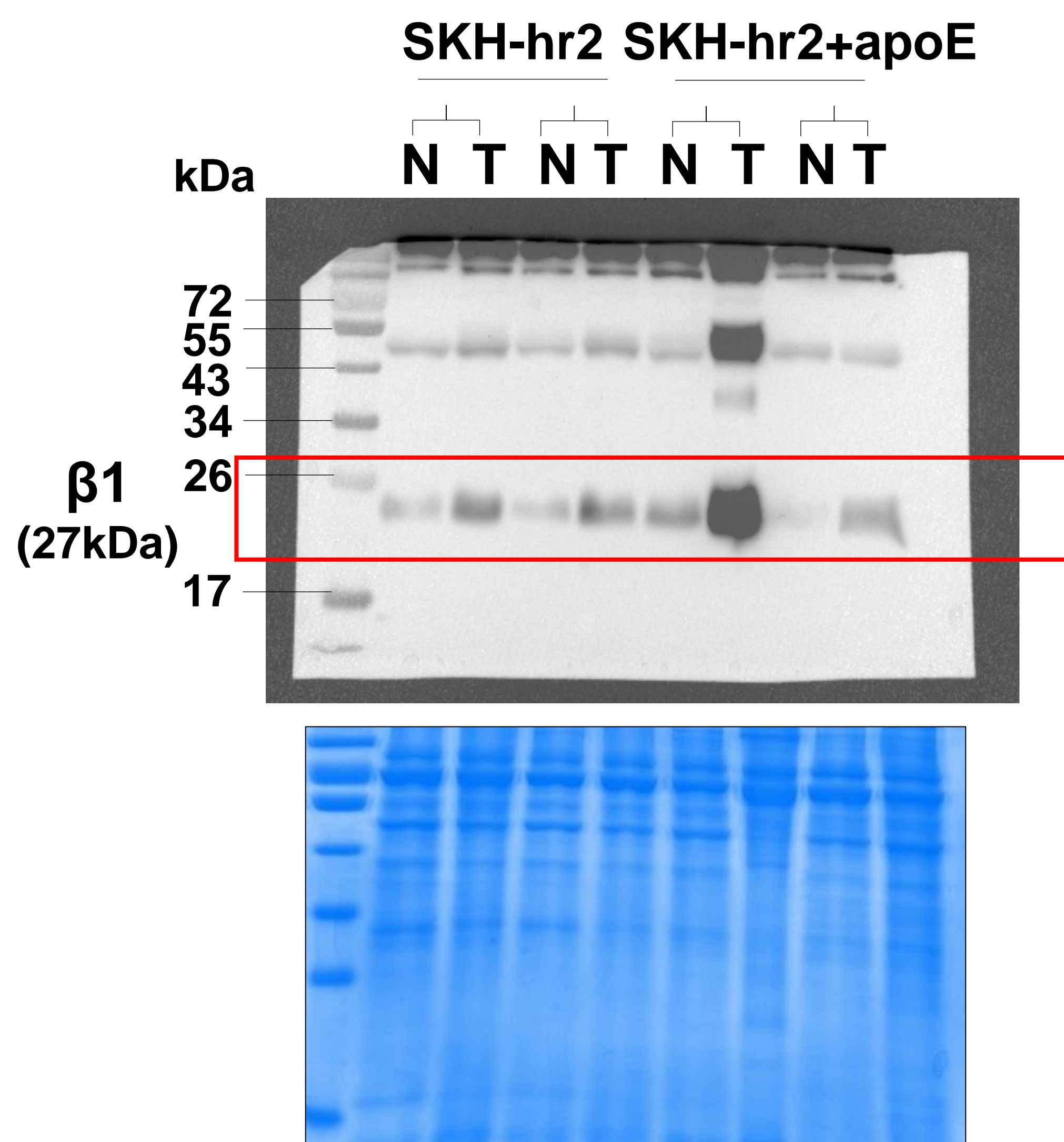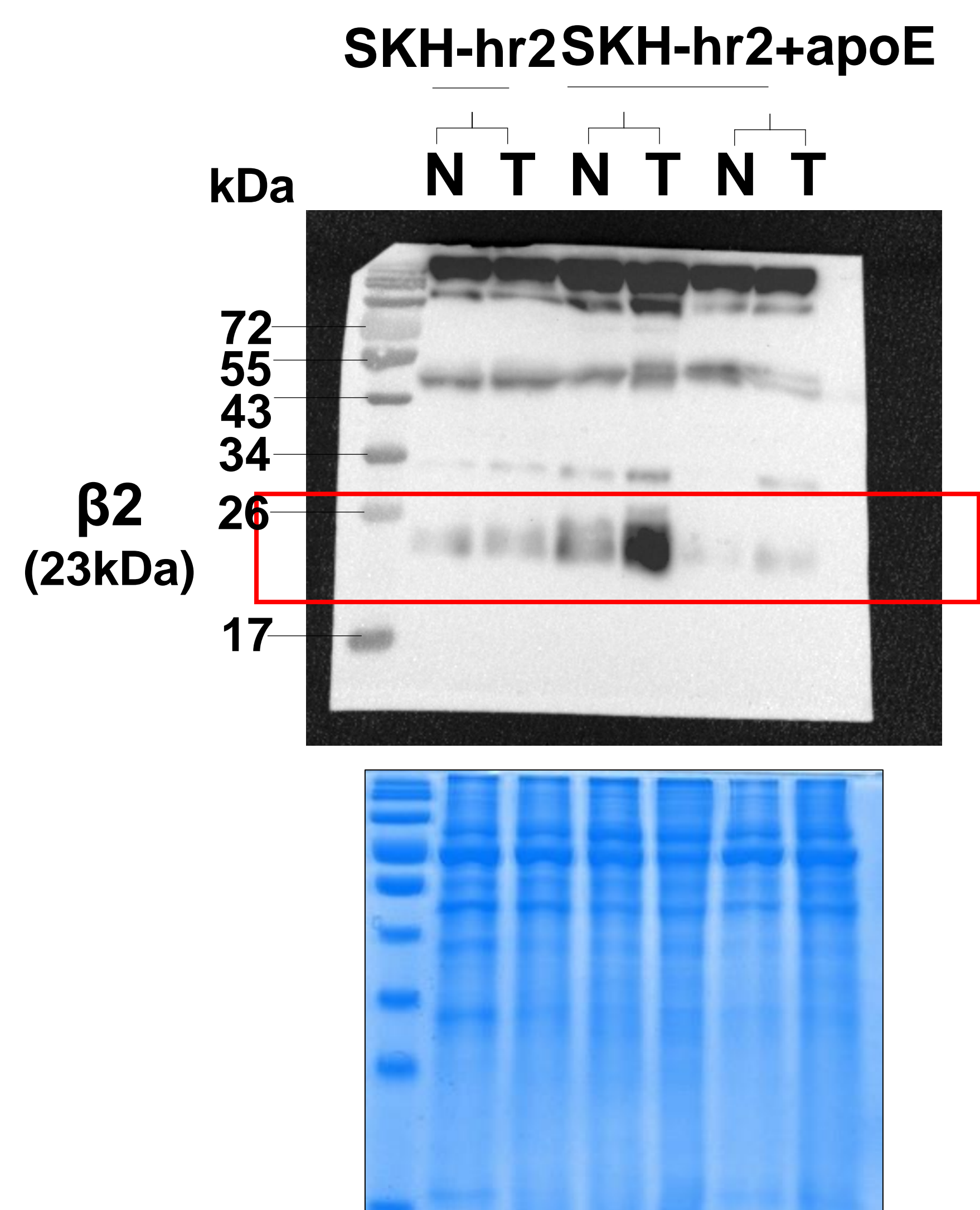

# SUPPLEMENTAL MATERIAL

Representative immunoblots of  $\beta 1$  and  $\beta 2$   
proteasome subunits

Supplement: Supplementary file 1 [file cancers-16-03546-s001.zip › cancers-3211309-supplementary.pdf]
